# Pneumoretroperitoneum, pneumomediastinum, and neck emphysema due to rectal diverticulosis

**DOI:** 10.1002/ccr3.6679

**Published:** 2022-12-05

**Authors:** Floreta Kurti, Viola Cala, Gentian Vyshka

**Affiliations:** ^1^ Service of Gastrohepatology University Hospital Center “Mother Theresa” Tirana Albania; ^2^ Service of Radiology University Hospital Center “Mother Theresa” Tirana Albania; ^3^ Biomedical and Experimental Department, Faculty of Medicine University of Medicine in Tirana Tirana Albania

**Keywords:** diverticulosis, neck swelling, pneumomediastinum, pneumoretroperitoneum

## Abstract

Intestinal diverticulosis is a chronic disorder that might present with acute symptoms, due to colonic perforation. Pneumoperitoneum and air bubbles spreading in different anatomical locations can be seen. These complications need careful consideration and, when appropriate, surgery, for an otherwise chronic condition that can be successfully treated through conservative measures.

## Graphical abstract

A 55‐year old Albanian female, under treatment for intestinal diverticulosis, suffered an increase in abdominal pain. Air bubbles were seen on CT reaching neck structures. An emergency surgery was decided with resection of the bleeding diverticulum.

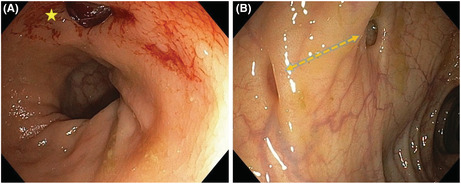

## 1 IMAGES IN CLINICAL MEDICINE

A 55‐year‐old Albanian female patient, under conservative treatment for chronic intestinal diverticulosis, noted a sudden increase in painful sensations in the lower abdomen, as well as an unexpected sense of suffocation. She had a previous colonoscopy two years before re‐appearing for consultancy, and the examination was repeated under anesthesia (midazolam, fentanyl).

**FIGURE 1:**
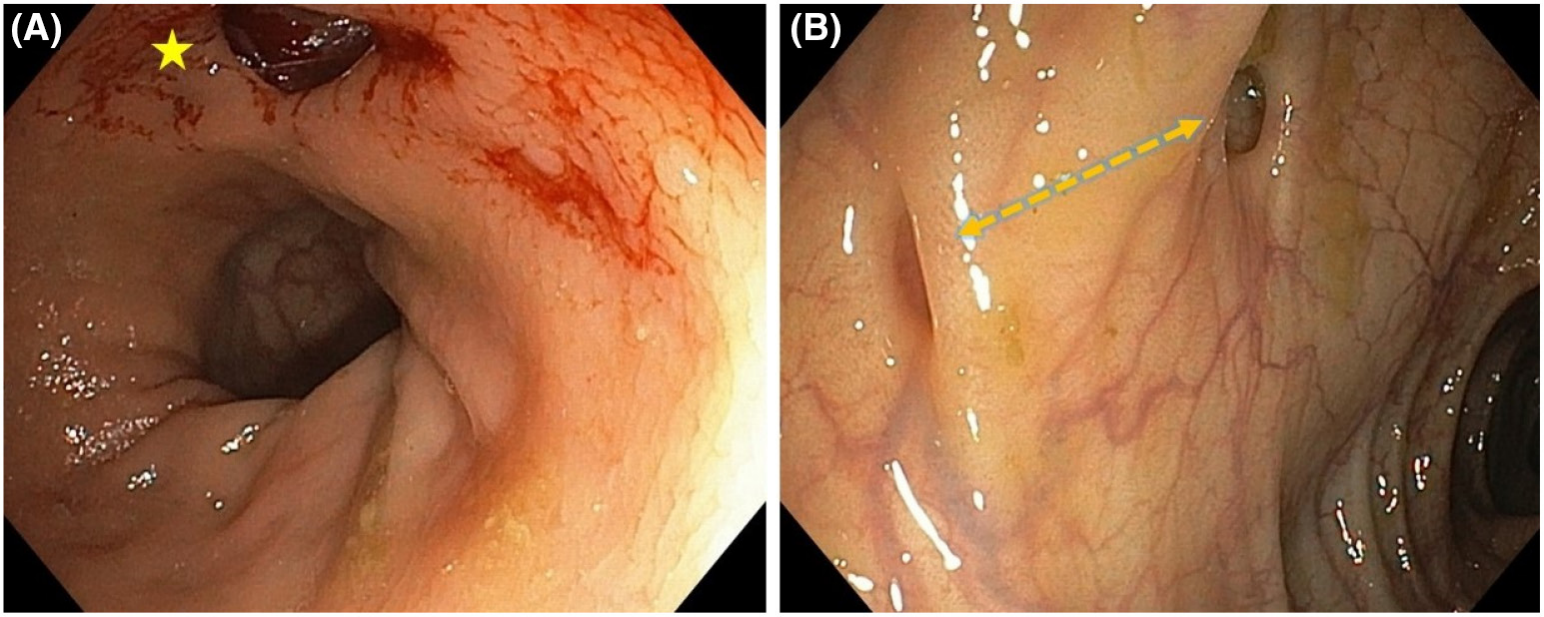
(A) Bleeding diverticulum twenty cm from the anal margin (star). (B) two other diverticula thirty centimeter orad from the first one (arrow).

Twenty centimeters from the anal margin a bleeding diverticulum approaching two centimeters in diameter was noted; some thirty centimeters orad other diverticula with normal mucosal appearance are seen (Figure 1).

**FIGURE 2:**
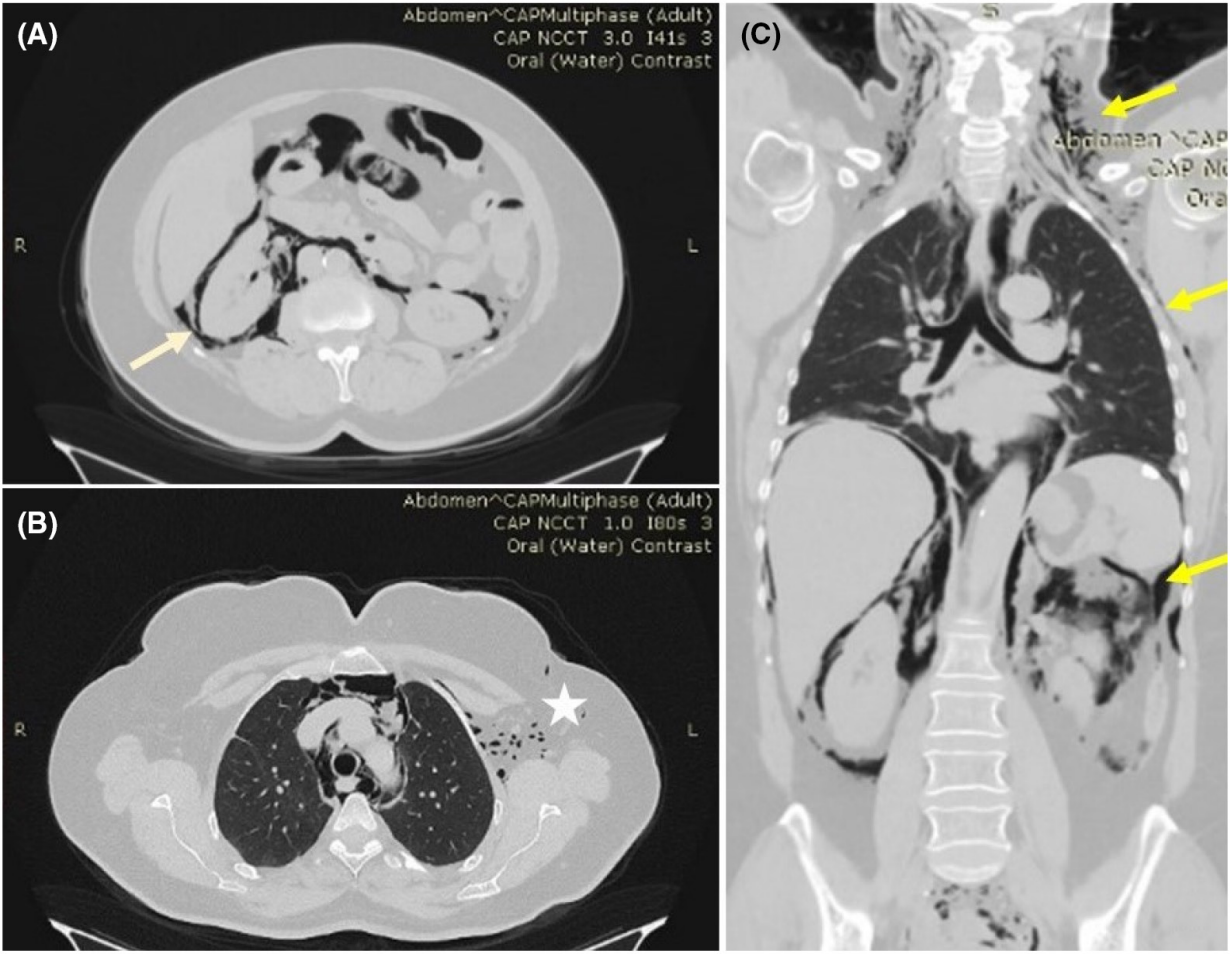
(A) Free perirenal air (arrow). (B) Subcutaneous air bubbles (star). (C) Neck emphysema (arrows).

The patient had the same afternoon a total body CT scan and an abdominal CT scan with oral contrast enhancement. Air bubbles were visible in both perirenal areas, advancing to the mediastinum and subcutaneously laterally, mostly in the left subaxillary and supraclavicular spaces, and reaching the neck structures (Figure 2).

The patient was sent for emergency surgical intervention, and she had a segmental resection of the interested area, anastomosis, and supportive postoperative treatment. Three months after the intervention during a follow‐up, she appeared healthy and free from complaints.

The occult perforation of diverticula is a frequent cause of the presence of free air in the abdomen, but cases of air bubbles reaching neck structures are reported as well.^1^, ^2^ Blunt trauma might play as well an important role, and its complications can reveal a previously undiagnosed diverticulosis.^3^ The actual position of considering diverticular disease mostly a chronic bowel disorder, that might be complicated acutely to the point of needing surgery, underscores the importance of close follow‐ups and multidisciplinary approach of the condition.^4^

## AUTHOR CONTRIBUTIONS

**Floreta Kurti:** Conceptualization; data curation; resources; writing – original draft. **Viola Cala:** Conceptualization; data curation; resources; writing – original draft. **Gentian Vyshka:** Conceptualization; data curation; methodology; validation; writing – review and editing.

## CONFLICT OF INTEREST

Nothing to declare.

## CONSENT

Written informed consent was obtained from the patient to publish this report in accordance with the journal's patient consent policy.

## Data Availability

Data sharing not applicable to this article as no datasets were generated or analysed during the current study.
